# Porcine endogenous retroviruses: an obstacle to cross during xenotransplantation

**DOI:** 10.1186/1471-2334-12-S1-O12

**Published:** 2012-05-04

**Authors:** Subramaniam Senthilkumar, Soma Guharthakurta, Kotturathu Mammen Cherian

**Affiliations:** 1 Frontier Lifeline Pvt. Ltd., Chennai, India

## Background

Xenotransplantation involves the transplantation of non-human tissue or organs to humans. Worldwide shortage of organs for clinical applications has shifted the focus towards non-human sources. Pig represents a rich source of organ donors but the presence of porcine endogenous retroviruses (PERVs) represents a particular risk and considered as a major obstacle during xenotransplantation. Among various types; PERV-C may recombine to form recombinant PERV-A/C and has the ability to infect human cells invitro and replicate at high titers. Our study aims to screen porcine tissue samples for provirus and virus particles of PERVs by PCR and reverse transcriptase PCR (RT-PCR).

## Methods

A total of 23 porcine heart tissue samples were included in this study. DNA and RNA from tissue samples were extracted using DNA and RNA extraction kits respectively. All the samples were subjected to standard PCR to detect pro-viral DNA and RT-PCR for mRNA expression of virus particle using specific primers.

## Results

All the 23 (100%) samples tested were positive for PERV-A and B pro-viral DNA by standard PCR, while 20/23 (87%) samples were positive for PERV-A and B RNA by RT-PCR. Fifteen out of 23 (65.2%) and 5/23 (21.7%) samples were positive for PERV-C and PERV-A/C pro-viral DNA respectively. Five out of 23 (21.7%) and 3/23 (13%) samples were positive for PERV-C and PERV-A/C RNA respectively.

## Conclusion

Low prevalence of PERV-C in our study indicates and paves a way to cross the obstacle in xeno-transplantation by using PERV-C free animal which may not produce infectious PERV-A/C recombinant virus.

